# Quantitative Sodium MRI of the Human Patellar Tendon

**DOI:** 10.1002/nbm.70404

**Published:** 2026-09-18

**Authors:** Rika Möller, Julia Jurisic, Lukas R. Drewes, Alexandra Ljimani, Hans‐Jörg Wittsack, Armin M. Nagel, Gerald Antoch, Lena M. Wilms, Anja Müller‐Lutz, Benedikt Kamp

**Affiliations:** ^1^ Department of Diagnostic and Interventional Radiology, Medical Faculty University Duesseldorf Duesseldorf Germany; ^2^ Institute of Radiology University Hospital Erlangen, Friedrich‐Alexander‐Universität Erlangen Nürnberg (FAU) Erlangen Germany; ^3^ Divison of Medical Physics in Radiology German Cancer Research Center (DKFZ) Heidelberg Germany; ^4^ Biomedical Physics Heinrich Heine University Düsseldorf Düsseldorf Germany

**Keywords:** ^1^H T_2_*, ^23^Na‐MRI, patellar tendinopathy, patellar tendon, sodium concentration, sodium MRI, sodium relaxation times

## Abstract

Sodium MRI can be used in musculoskeletal imaging to assess tissue composition because sodium concentration is related to glycosaminoglycan (GAG) content. In tendinopathy, the GAG content is elevated, resulting in a high signal in sodium MRI. Because biochemical changes typically precede structural alterations, sodium MRI may be a useful indicator for detecting early tendon matrix changes associated with tendinopathy. Quantitative sodium concentration determination has only been applied to Achilles tendons but not yet to smaller tendons, such as the patellar tendon. Due to its smaller diameter, the patellar tendon is more susceptible to partial volume artifacts, making accurate sodium concentration quantification more challenging. However, partial volume correction (PVC) may help overcome these difficulties. In this study, we determined the apparent tissue sodium concentration (aTSC) in seven patients with patellar tendinopathy and 20 healthy controls using clinical 3 T MRI with a density‐adapted 3D radial MR‐sequence. Sodium images were corrected for partial volume effects using an in‐house developed PVC, called the estimated single target correction (eSTC). Additionally, ^1^H T_2_* values were calculated for comparison, as they are sensitive to structural alterations. Sodium relaxation times were determined in three healthy controls, resulting in a T_1_ of 30.2 ± 0.4 ms and a biexponential T_2s_* of 0.7 ± 0.2 ms and T_2l_* of 13.0 ± 0.1 ms. The short component *p*_*s*_ accounted for 35.7% ± 2.1% of the transversal relaxation. aTSC values were 35.4 ± 10.1 mM in tendinopathy patients and 21.6 ± 3.9 mM in healthy controls. ^1^H T_2_* values were 5.4 ± 1.9 ms and 2.8 ± 0.3 ms, respectively. Differences between patients and controls were significant for both parameters. These findings support the suitability of sodium MRI for quantitative assessment of the patellar tendon and demonstrate its potential for visualizing biochemical changes associated with patellar tendinopathy.

## Graphical abstract

Sodium MRI and ^1^H T_2_* measurements of the patellar tendon were conducted in 20 healthy volunteers and seven tendinopathy patients at a clinical field strength. Significant differences between the two groups were observed with both parameters. This demonstrates the feasibility of using sodium MRI in the patellar tendon and the potential for early diagnosis of patellar tendinopathy in the future.

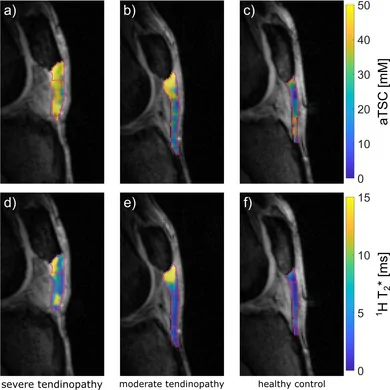

## 1 Introduction

Patellar tendinopathy is a common sports injury with a prevalence in jumping sports such as volleyball, basketball, or football [1, 2, 3]. Passive treatment with exercises is often employed after diagnosis; however, limited success has been found [4], and many patients experience long‐term restrictions, even years after the initial injury [5]. Early intervention could halt injury progression [6], but it is seldom diagnosed before late‐stage symptoms occur [6, 7], when treatment becomes more difficult [8]. It has been shown that exercises performed after a 2‐week rest period are the most effective treatment [9]. During this rest period, no macroscopic changes or changes in the collagen matrix occur. However, the concentration of glycosaminoglycans (GAGs) increases after overuse, which changes the microstructural tension and the viscoelastic properties, likely initiating a healing response [6].

Diagnosis involves physical examination and imaging techniques such as ultrasound and MRI [10, 11]. In MRI, patellar tendinopathy can be characterized by tendon thickening and, in most cases, a heightened signal intensity in proton‐density‐weighted images, often in the proximal part of the tendon [10]. These methods can depict structural changes, but they are limited in their ability to detect the biochemical changes that characterize early disease stages [12]. Sodium MRI is a functional imaging technique sensitive to biochemical changes in tissues, including increased GAG content. In musculoskeletal imaging, it has predominantly been used to investigate cartilage, muscles, and the spine [13].

In tendons, sodium MRI has mostly been applied to the Achilles tendon, where both sodium signal‐to‐noise ratios (SNRs) [14, 15] and apparent tissue sodium concentrations (aTSCs) [16] have been measured in vivo [14, 15, 16] and ex vivo [12, 14]. The correlation between GAG content and sodium MRI has been demonstrated ex vivo [12], and significant differences in sodium SNR between healthy volunteers and patients with Achilles tendinopathy have been reported [14]. Additionally, Marik et al. [17] applied sodium MRI to the patellar tendon for the first time at 7 T in healthy volunteers and patients with type 1 diabetes. Although the patients did not exhibit morphological changes in the tendon, they had a significantly higher sodium signal. However, they did not calculate aTSCs and examined three slices in the middle part of the tendon. Because patellar tendinopathy is most prevalent in the proximal part of the tendon [10], measurement techniques that include this section would be preferable.

Sodium MRI is limited by the low concentration of ^23^Na in vivo, resulting in a reduced SNR, limited spatial resolution, and increased partial volume artifacts [18]. Furthermore, radial acquisition schemes are often used to achieve shorter TEs for sodium MRI [19], as transversal sodium relaxation times are especially short [18]. However, compared with Cartesian acquisition schemes, radial acquisitions lead to stronger blurring with the point spread function (PSF) [20, 21, 22]. Therefore, small structures such as tendons are difficult to differentiate from their surroundings. Because the Achilles tendon is the thickest tendon in the human body [23], sodium MRI is more applicable to it than to other tendons. Yet, it remains substantially affected by partial volume effects (PVEs) [24]. Smaller tendons, like the patellar tendon [25], are expected to be affected even more. With an average thickness of 2–3 mm [26], the patellar tendon is about half as thick as the 6‐mm‐thick Achilles tendon [27]. The application of partial volume corrections (PVCs) [24, 28] can improve the effective resolution and enable measurements of smaller tendons, such as the patellar tendon.

This study aims to determine aTSCs and sodium relaxation times in the entire patellar tendon at a clinical 3 T field strength. Sodium relaxation times were used to correct relaxation weighting when calculating aTSC values. PVEs were corrected with an in‐house developed PVC called the estimated single target correction (eSTC) [24]. Additionally, we aimed to determine proton ^1^H T_2_* values for comparison, because they have been used in previous studies to characterize patellar tendons and patellar tendinopathy [29, 30, 31]. We hypothesized that sodium imaging protocols originally developed for the Achilles tendon can be adapted for the patellar tendon and that both aTSC and ^1^H T_2_* values are increased in patients with patellar tendinopathy.

## 2 Experimental Section

### 2.1 Study Design and Population

Twenty healthy volunteers (eight female, 12 male, mean age 28.3 ± 5.6 years) and seven patients with patellar tendinopathy (all male, mean age 34.0 ± 11.4 years) were examined in this prospective study. All images of the healthy controls were obtained from the right knee. Three healthy volunteers were additionally imaged for the determination of sodium relaxation times. Participants were excluded if other pathologies in the region surrounding the patellar tendon were reported or detected during clinical evaluation. Participants were included in the patient cohort if they had a previous diagnosis of patellar tendinopathy or presented with chronic knee pain and had tendinopathy confirmed by a radiologist (A.L., with 10 years of experience in musculoskeletal imaging) based on a standard clinical MRI protocol. The severity of tendinopathy can vary among patients, depending on how much of the tendon is affected. To descriptively categorize these differences, the patients were classified as having minimal, moderate, or severe tendinopathy based on structural alterations observed in the clinical images. Written consent was obtained from all participants, and ethics approval was received from the Ethics Committee, Medical Faculty of the Heinrich‐Heine‐University Düsseldorf (study number 2021‐1393_1).

### 2.2 Image Acquisition

Images were acquired at a clinical 3 T MRI (Siemens MAGNETOM Prisma, Siemens Healthineers, Erlangen, Germany) with a bore size of 60 cm and XA 60 software. The maximum possible gradient amplitude was 80 mT/m with a slew rate of 200 T/m/s.

For subsequent transmit/receive inhomogeneity corrections, a sensitivity profile was created to characterize the surface coil by measuring a cylindrical phantom containing a homogenous sodium chloride (NaCl) solution with a concentration of 154 mM. The phantom had a diameter of 18 cm and a height of 11 cm. Two additional rectangular phantoms containing the same NaCl solution were placed behind the coil. These phantoms had dimensions of 23 cm by 13 cm by 6 cm. These phantoms were imaged using the acquisition parameters of the “coil sensitivity” protocol listed in Table 1, averaging 20 times to minimize noise.

**TABLE 1:** Acquisition parameters of the DA‐3D‐RAD sequence for determination of the coil sensitivity using a homogeneous phantom, sodium T_1_ and T_2_* relaxation times determination, sodium concentration determination, and ^1^H imaging for segmentation and ^1^H T_2_* determination.

	Coil sensitivity	^23^Na T_1_	^23^Na T_2_*	^23^Na Concentration	T_2_* ^1^H Imaging
Sequence	DA‐3D‐RAD	DA‐3D‐RAD	DA‐3D‐RAD	DA‐3D‐RAD	DA‐3D‐RAD
Nucleus	^23^Na	^23^Na	^23^Na	^23^Na	^1^H
Orientation	Sagittal	Sagittal	Sagittal	Sagittal	Sagittal
Repetition time (ms)	15	8/9/10/15/30	30	20	12
Echo time (ms)	0.3	0.1	[0.1/5.9/11.8/17.6] [1.5/7.3/13.2/19.0] [3.0/8.8/14.7/20.5]	0.1	0.1/3.0/6.0/9.0
Field of view (mm^3^)	180 × 180 × 180	180 × 180 × 180	180 × 180 × 180	180 × 180 × 180	180 × 180 × 180
Projections	50,000	50,000	40,000	50,000	25,000
Resolution (mm^3^)	2 × 2 × 2	2 × 2 × 2	2 × 2 × 2	2 × 2 × 2	1 × 1 × 1
Flip angle (°)	90	90	90	90	5
RF Pulse duration (ms)	0.50	0.16	0.16	0.16	0.16
Readout time (ms)	5	5	5	5	1
Averages	20	1	1	1	1
Spoiler gradient amplitude (mT/m)	40	40	40	40	40
Spoiler gradient slew rate (T/m/s)	180	180	180	180	120
Total scan time (h:min:s)	4:10:00	1:00:00	1:00:00	00:16:40	00:05:00

All participants underwent imaging with two coils: a dual‐tuned ^23^Na/^1^H surface coil (RAPID Biomedical GmbH, Rimpar, Germany) for sodium images and proton images for segmentation, as well as a ^1^H 15 channel knee coil (Tx/Rx Knee 15 Flare Coil, Siemens Healthineers, Erlangen, Germany) for clinical reference images. Sodium and proton images with the surface coil were acquired using a density‐adapted 3D radial sequence (DA‐3D‐RAD) [19]. The volunteers were positioned supine, feet first, with a small foam cushion placed beneath the knee to taut the patellar tendon while the coil was placed on the knee. 3D‐printed props were placed on either side of the knee to support the coil. To facilitate the aTSC calculation, four reference phantoms (1.0 cm in diameter and 3.5 cm in height) with known sodium concentrations were placed behind the coil. The phantoms contained 4% agarose content by weight (ROTI Agarose, Carl Roth GmbH & Co. KG, Karlsruhe, Germany) with sodium concentrations of 25, 50, 75, and 100 mM. Initially, a flip angle calibration was conducted for all ^1^H and ^23^Na images with the dual tuned coil. This calibration used TE = 0.35 ms, TR = 1500 ms, and a rectangular RF‐pulse with a 0.5‐ms duration. Additionally, a manual B_0_ shim was performed using the vendor provided ^1^H‐B_0_ shimming routine.

To facilitate relaxation correction, sodium relaxation times were measured in three healthy volunteers. T_1_ and T_2_* relaxation times were acquired in separate imaging sessions, because both protocols had a long examination time. For both measurements, ^1^H images for segmentation were acquired with the DA‐3D‐RAD sequence and the dual‐tuned coil. T_1_ measurements were conducted with a short TE of 0.1 ms, five different repetition times with a maximum TR of 30 ms and 50,000 projections. This resulted in a total acquisition time of 1:00 h:min. The T_2_* relaxation times were acquired with 12 different echo times with a minimum TE of 0.1 ms, a maximum TE of 20.5 ms, a TR of 30 ms, and a reduced number of 40,000 projections to keep the measurement time reasonable. Three sequences with four echo times each were acquired separately with interleaved echo times to facilitate a closer sampling of the transversal relaxation. This also resulted a total acquisition time of 1:00 h:min.

The imaging protocol for determining aTSC values was chosen to maximize SNR at an acceptable measurement time of 16:40 min:s. Additionally, ^1^H‐images were acquired with four different echo times with the DA‐3D‐RAD to facilitate proton T_2_* determination. To assess short‐term reproducibility, this protocol was repeated on three healthy volunteers after a brief break and repositioning in the scanner. All imaging parameters with the ^23^Na/^1^H surface coil are listed in Table 1.

Following the acquisition with the surface coil, the participants were repositioned in the knee coil for clinical evaluation. Three proton‐density‐weighted sequences, one sagittal T_1_‐weighted sequence, and one coronal T_2_‐weighted sequence were acquired. Additionally, a 3D double echo steady state (DESS) sequence was acquired. The imaging parameters for imaging with the ^1^H 15 channel knee coil are summarized in Table 2.

**TABLE 2:** Image acquisition parameters of the protocol for clinical evaluation. All sequences are Siemens standard sequences for knee imaging. The transversal PD‐weighted fat‐saturated (fs) sequence was angled perpendicular to the patellar tendon, and the coronal T2‐weighted sequence was aligned parallel to the patellar tendon.

Sequence	PD‐weighted fs	PD‐weighted fs	T1‐weighted	T2‐weighted	T2‐weighted 3D
Sequence type	TSE	TSE	TSE	TSE	DESS
Turbo factor	10	10	3	12	—
PIAF	2	2	2	2	2
Orientation	Sagittal	Transversal/transversal angled	Sagittal	Coronal angled	Sagittal
Repetition Time (ms)	4980	5070	864	3380	14.7
Echo time (ms)	42	40	13	85	5.04
Field of View (mm)	160 × 160	160 × 160	160 × 160	160 × 160	160 × 160
Pixel size (mm)	0.3 × 0.3	0.3 × 0.3	0.3 × 0.3	0.4 × 0.4	0.6 × 0.6
Flip angle (°)	180	180	180	180	25
Slices	35	35	35	19	176
Slice gap (%)	10	10	10	10	20
Slice thickness (mm)	3	3	3	2	0.6
Examination time (min:s)	3:19	3:23 + 3:23	3:10	1:41	3:40

Abbreviations: DESS, double echo steady state; fs, fat saturated; PD, proton‐density; PIAF, parallel imaging acceleration factor; TSE, turbo spin echo.

### 2.3 Partial Volume Correction

Radial acquisition sequences are especially susceptible to PVEs, due to PSF smearing [22, 28]. These artifacts were corrected using an in‐house developed PVC called the eSTC [24]. It uses a simulated PSF of the DA‐3D‐RAD sequence to calculate region spread functions (RSF) of the tendon, surroundings and phantoms [28]. The simulated PSF depends primarily on the k‐space trajectory of the sequence [22] and the tissue sodium relaxation times [20]. Mixed signals due to spill‐in were corrected by estimating the true signal and convolving the estimated signal with its PSF. Then, the signal contribution to adjacent structures can be defined and subtracted. After removing the spilled‐in signal, the spilled‐out signal can be recovered by dividing by its own RSF. The result is a corrected signal distribution, *C*_*i*_, where the spilled‐in contribution is defined by the estimated signal, *E*_*j*_, from the adjacent structure, convolved with its PSF (*PSF*_*j*_), inside the targets mask, *m*_*i*_. The spilled‐out signal is then recovered through RSF division:

(1) $$C_{i}=\frac{1}{\mathrm{RS}F_{i}}\left[S_{i}-\sum_{\begin{array}{c} i\\ j\neq i \end{array}}m_{i}\cdot \left(E_{j}*\mathrm{PS}F_{j}\right)\right]$$

When the surrounding signal is equal to zero, as is the case with the reference phantoms, the sum in Equation (1) is also zero, making it a simple RSF division [24].

### 2.4 Image Post‐Processing

All DA‐3D‐RAD images were externally reconstructed with a MATLAB script (MATLAB, The MathWorks Inc. Natick, United States, R2022a) using a nonuniform fast Fourier transformation (NUFFT) and a Hamming Filter to reduce Gibbs ringing [19]. The sodium images were zerofilled in all three dimensions to match the resolution of the proton images, resulting in a 180 × 180 × 180 matrix size for all images. Because movement is to be expected during the long acquisition times of the relaxation measurements, the sodium images of those imaging sessions were motion corrected using an in‐house developed software called stroketool and a cross‐correlation algorithm based on advanced normalization tools for image registration [32, 33]. Regions of interest (ROIs) were drawn on the DA‐3D‐RAD ^1^H‐images with the software ITK‐snap (version 3.8.0, Cognitica, Philadelphia, PA, United States) [34]. ROIs were defined to encompass the entire patellar tendon. To assess variability along the tendon, the tendon ROI was additionally divided into three equal parts along its longitudinal axis, corresponding to the proximal, middle, and distal tendon regions. One ROI was placed for each reference phantom. For intra‐reader reliability assessment, the tendon ROIs were drawn twice by the same reader R.M. (with 4 years of experience in musculoskeletal imaging) with an interval of at least 2 weeks between the two segmentations. For inter‐reader reliability, J.J. (with 1 year of experience in musculoskeletal imaging) drew the ROIs once under the supervision of L.W. (with 10 years of experience in musculoskeletal imaging). All subsequent analyses were conducted with in‐house developed MATLAB scripts (MATLAB, The MathWorks Inc., Natick, United States, R2022a).

For the sodium relaxation time analysis, the mean signal intensities across the tendon and reference phantom ROIs were calculated and fitted for T_1_ according to

(2) $$S\left(\mathrm{TR}\right)=S_{0}\left(1-e^{-\frac{\mathrm{TR}}{T_{1}}}\right)+\text{noise}$$

where *S* (*TR*) is the mean sodium signal at the repetition time *TR* and *S*_0_ is the maximum signal after excitation. The T_2_* times were fitted biexponentially with a short component T_2s_* and long component T_2l_* as well as the fraction of the short transversal relaxation time *p*_*s*_ [35]:

(3) $$S\left(\mathrm{TE}\right)=S_{0}\left(p_{s}\;e^{-\frac{\mathrm{TE}}{T_{2s}^{*}}}+\left(1-p_{s}\right)e^{-\frac{\mathrm{TE}}{T_{2l}^{*}}}\right)+\text{noise}$$

With *S* (*TE*) as the mean sodium signal at the echo time *TE*.

The ^1^H T_2_* maps of the patellar tendon were calculated by fitting voxel‐wise monoexponentially with a noise offset as a free parameter according to

(4) $$S\left(\mathrm{TE}\right)=S_{0}\;e^{-\frac{\mathrm{TE}}{T_{2}^{*}}}+\text{noise}$$

For the aTSC determination, an additional ROI encompassing the tendon's surroundings was defined for PVC. This ROI encompassed all sodium‐containing tissues surrounding the tendon within the coil's sensitivity, primarily including the skin, fat, and cartilage, whereby fat directly surrounds the tendon. Because to date there are no fat sodium relaxation times in the literature, the relaxation times for muscle [36] were used for the PSF simulation [37] of the surrounding ROI, while the previously measured relaxation times were used for the tendons and the reference phantoms PSFs. After PVC, the sodium images were corrected for coil sensitivity by manually registering the normalized sensitivity profile to each image. The registered sensitivity profile was then inversed to create weighting factors that corrected for inhomogeneous coil sensitivity, which were multiplied onto the respective sodium image of the knee. The mean signal intensities of each ROI were calculated, and those of the four reference phantoms were fitted linearly with the known concentrations. From the resulting slope and intercept, the tissue concentrations were calculated. Although agarose is relatively close to tissue relaxation times, there are still relevant differences to actual tissue that can influence the aTSC determination [38]. Therefore, a weighting factor was calculated from the then known relaxation times of the previously measured volunteers and applied to the aTSC calculation [16, 38]. In addition to the mean values, aTSC maps were calculated for each dataset.

The reproducibility was quantified by calculating the percentage deviation Δ of the results of the second measurement *S*_*M*2_ from the first measurement *S*_*M*1_ of each ROI according to

(5) $$\Delta =\left|\frac{\left(S_{M1}-S_{M2}\right)}{S_{M1}}\right|$$

### 2.5 Statistics

The statistical evaluation was conducted in python 3.12 using the SciPy 1.17.1 library [39]. To evaluate the differences in aTSCs and ^1^H T_2_* relaxation times within the tendon, the mean values in the proximal, middle, and distal section were tested individually for patients and healthy controls for significant differences using a Bonferroni‐corrected a Wilcoxon test. Differences between patients and healthy controls were evaluated using a two‐sided Mann–Whitney *U* test. To assess intra‐ and inter‐reader reliability, the two‐way random effects single‐measure correlation coefficient for absolute agreement (ICC2) [40] was calculated individually for patients and healthy volunteers, as the ICC is influenced by how similar the results are to each other. The ICC of an inhomogeneous cohort is more likely to be overestimated [41]. Because patients and volunteers can be considered dissimilar due to the pathology, the ICCs were calculated separately to avoid overestimation. The ICCs were categorized according to Koo et al.’s classification [42].

## 3 Results

**FIGURE 1:**
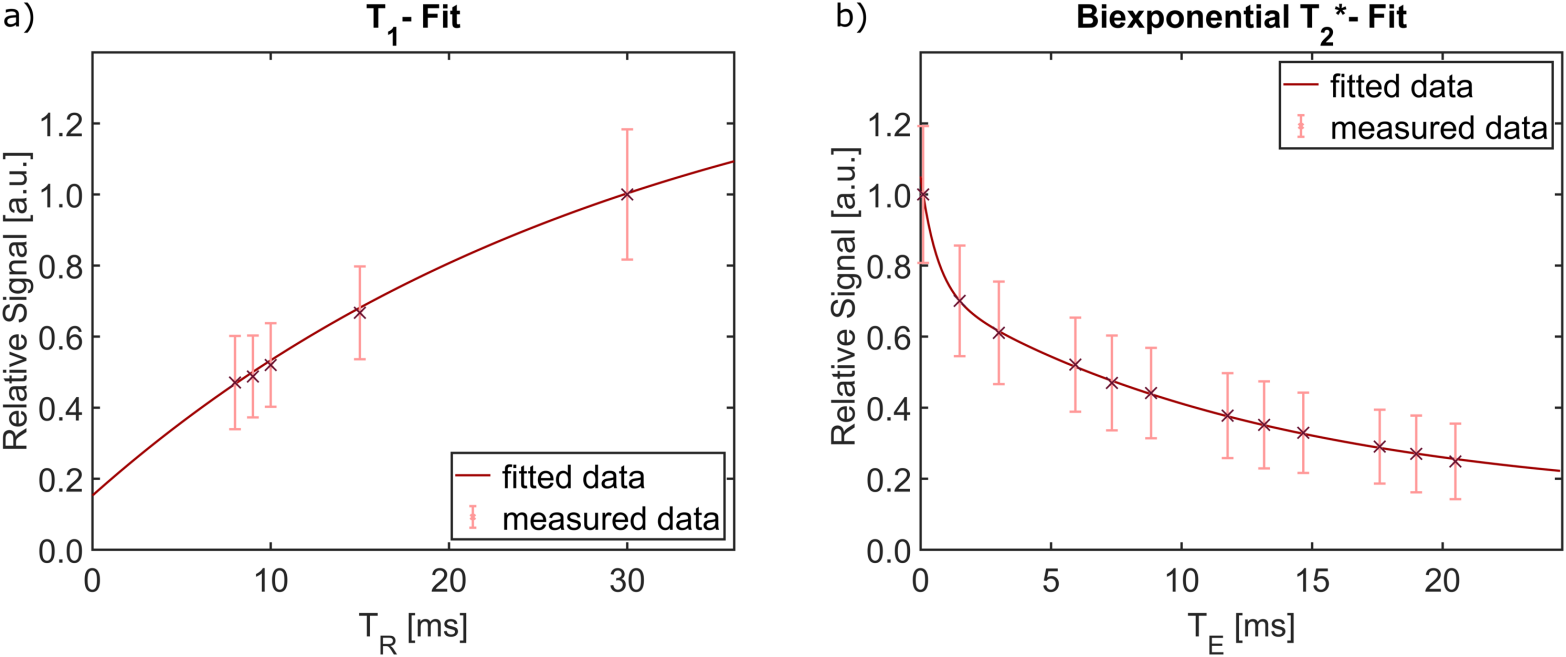
Exemplary ^23^Na T_1_ (a) and biexponential T_2_* fits (b) of the healthy patellar tendon of one volunteer. The results of the fits shown here were (a) ^23^Na T_1_ = 30.3 ms, *R* ^2^ = 0.9971, and (b) ^23^Na T_2s_* = 0.63 ms, ^23^Na T_2l_* = 13.0 ms, *p*_*s*_ = 33.9%, *R* ^2^ = 0.9996.

Sodium relaxation times were determined for the patellar tendon and the reference phantoms in three healthy volunteers resulting in a mean T_1_ of 30.2 ± 0.4 ms, T_2s_* of 0.7 ± 0.2, and T_2l_* 13.0 ± 0.1 ms. The results are summarized in Table 3. An exemplary fit for both the T_1_ and T_2_* measurements of the tendon is shown in Figure 1.

**TABLE 3:** Sodium T_1_ and biexponential T_2_* relaxation times of three healthy controls and the 4% agarose reference phantoms.

Region	^23^Na T_1_ (ms)	*R* ^2^ (^23^Na T_1_)	^23^Na T_2s_* (ms)	^23^Na T_2l_* (ms)	*p*_*s*_ (%)	*R* ^2^ (^23^Na T_2_*)
Tendon	30.2 ± 0.4	0.9954 ± 0.0012	0.7 ± 0.2	13.0 ± 0.1	35.7 ± 2.1	0.9989 ± 0.0005
100 mM phantom	38.6 ± 1.0	0.9990 ± 0.0006	5.5 ± 0.9	11.0 ± 0.7	63.2 ± 0.7	0.9903 ± 0.0083
75 mM phantom	38.9 ± 1.0	0.9989 ± 0.0004	6.3 ± 0.6	11.3 ± 0.1	64.5 ± 0.9	0.9933 ± 0.0035
50 mM phantom	39.5 ± 1.7	0.9923 ± 0.0046	5.4 ± 0.7	11.4 ± 0.2	64.6 ± 2.2	0.9863 ± 0.0090
25 mM phantom	43.4 ± 2.2	0.9922 ± 0.0030	4.7 ± 0.7	11.1 ± 0.7	64.3 ± 1.2	0.9759 ± 0.0124
Phantom mean	40.1 ± 1.9	0.9956 ± 0.0033	5.5 ± 0.6	11.2 ± 0.2	64.1 ± 0.5	0.9865 ± 0.0066

Abbreviations: *p*_*s*_, fraction of T_2s_* of the total T_2_* relaxation; *R* ^2^ coefficient of determination for fits.

**FIGURE 2:**
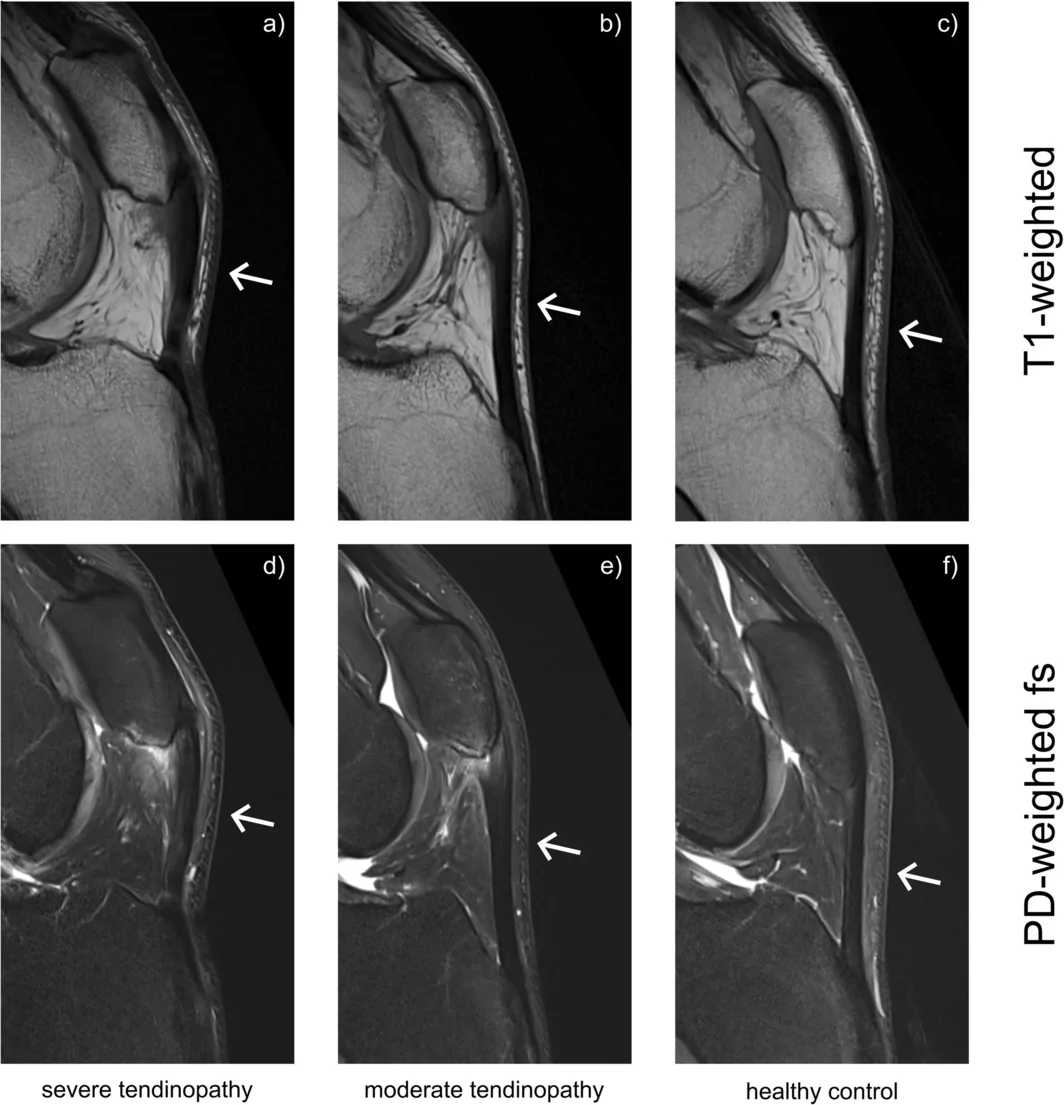
T1‐weighted and fat‐saturated PD‐weighted clinical standard images of a patient with tendinopathy with severe structural alterations (a, d), a tendinopathy patient with moderate structural alterations predominantly in the proximal section (b, e), and a healthy control (c, f). The patellar tendon is indicated with a white arrow.

Seven participants showed structural alterations in the clinical imaging protocol indicating patellar tendinopathy. The severity ranged from minimal to severe structural alterations, and the affected section of the tendon was, in some cases, confined to the proximal region and, in others, spanned the entire tendon (Figure 2).

**FIGURE 3:**
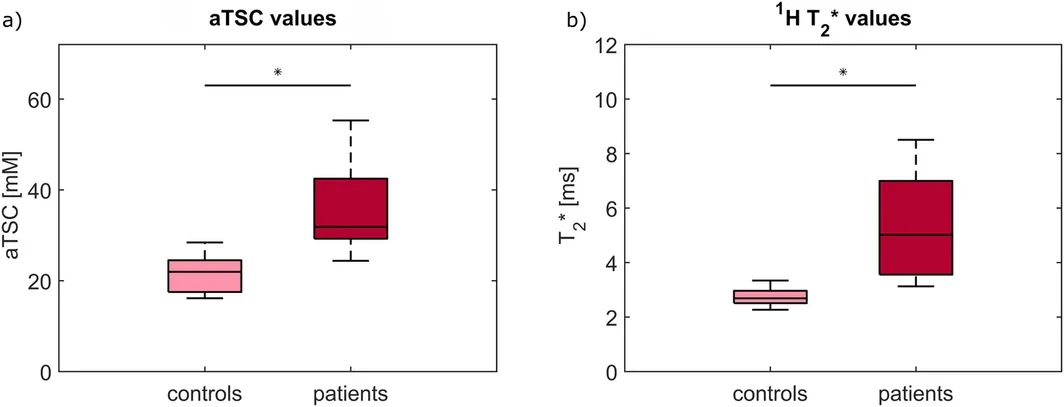
Boxplots of mean aTSC (a) and ^1^H T_2_* (b) values of the entire patellar tendon of seven patients with patellar tendinopathy and 20 healthy controls. The differences between controls and patients were significant with both parameters (*).

**FIGURE 4:**
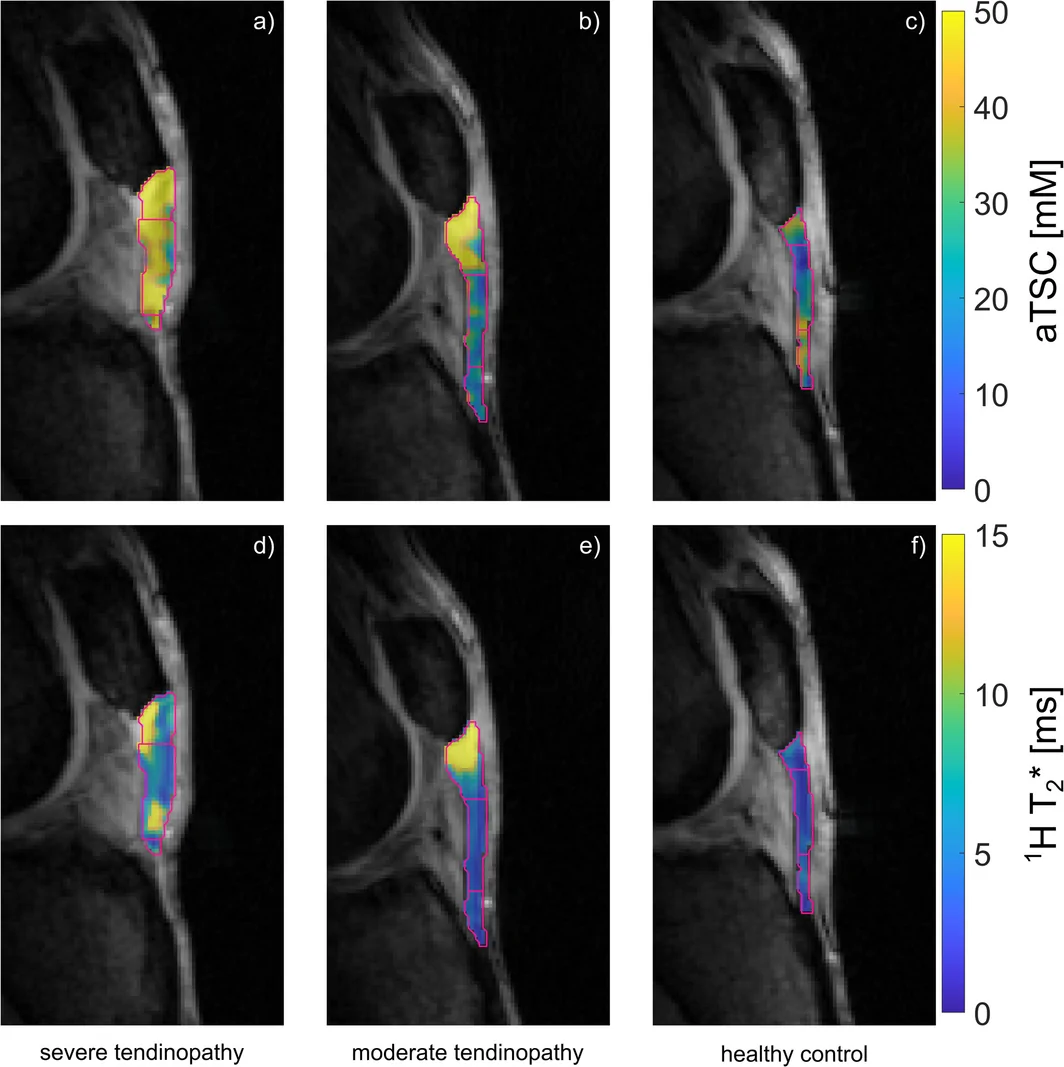
aTSC and ^1^H T2* maps of a tendinopathy patient with severe structural alterations (a, d), a tendinopathy patient with moderate structural alterations predominantly in the proximal section (b, e), and a healthy control (c, f). The division of the tendon ROI into the proximal, middle, and distal sections is outlined. Note that the distal section in (a) and (d) seems small in this image due to the angle of the knee perpendicular to the image slice.

aTSC values were determined in healthy controls and patients, resulting in mean aTSC values of 21.6 ± 3.9 mM and 35.4 ± 10.1 mM, respectively. Across the healthy tendon, the aTSC values were relatively evenly distributed with a slight elevation at the endpoints. This elevation was marginally significant (*p* = 0.046) between the proximal and middle sections. There were no significant differences between the tendon sections in patients. The aTSC values were significantly higher in tendinopathy patients than in healthy controls, both in the whole tendon (Figure 3a)) and in the individual sections. ^1^H T_2_* values showed a significant difference, with mean relaxation times of 2.8 ± 0.3 ms in healthy controls and 5.4 ± 1.9 ms in patients (Figure 3b)). The aTSC and ^1^H T_2_* results for all tendon sections are listed in Table 4, and exemplary aTSC and ^1^H T_2_* maps are shown in Figure 4.

**TABLE 4:** Results for aTSC and ^1^H T_2_* values of the patellar tendon of 20 healthy controls and seven patients with patellar tendinopathy for the proximal, middle, and distal part of the tendon, as well as for the whole tendon. The parameters were tested for significant differences between patients and healthy controls with a two‐tailed Mann–Whitney *U*‐test.

Parameter	Tendon section	Controls	Patients	*p*
aTSC (mM)	Proximal	22.7 ± 4.9	40.1 ± 10.3	< 0.001
	Middle	20.2 ± 4.8	30.9 ± 11.0	0.013
	Distal	23.0 ± 6.1	35.1 ± 12.0	0.011
	Total	21.7 ± 3.9	35.5 ± 10.1	< 0.001
^1^H T_2_* (ms)	Proximal	2.7 ± 0.3	6.0 ± 2.3	< 0.001
	Middle	2.7 ± 0.3	4.9 ± 2.5	0.004
	Distal	2.9 ± 0.5	4.6 ± 2.3	0.009
	Total	2.8 ± 0.3	5.4 ± 1.9	< 0.001

Abbreviation: aTSC, apparent tissue sodium content.

Intra‐reader analysis of aTSCs yielded ICCs of ≥ 0.98 for patients and ≥ 0.96 for healthy controls. For ^1^H T_2_* values, the ICCs were > 0.99 in patients and ≥ 0.96 in healthy controls. Similar values were obtained in the inter‐reader analysis with > 0.99 in patients and ≥ 0.92 in healthy controls for aTSC values, and for ^1^H T_2_*, ≥ 0.97 in patients and ≥ 0.98 in healthy controls. All parameters are indicative of excellent intra‐ and inter‐reader reliability (ICC > 0.90) [42]. To assess the short‐term reproducibility, the measurement was repeated with three subjects after repositioning and the deviation between the measurements was calculated. The mean total deviation of aTSC values was 4.1% ± 0.7% and 9.5% ± 5.5% in ^1^H T_2_* values. The results are listed in Table 5.

**TABLE 5:** Percentage deviation (Δ) between two consecutive measurements of three healthy volunteers after repositioning (Equation 5) for aTSC and ^1^H T_2_* values. The deviation was calculated for the mean values of each section of the tendon, as well as the whole tendon.

Parameter	Δ Proximal (%)	Δ Middle (%)	Δ Distal (%)	Δ Total (%)
aTSC	5.5 ± 4.9	4.1 ± 3.2	8.6 ± 5.8	4.1 ± 0.7
^1^H T_2_*	9.8 ± 4.3	7.0 ± 3.8	12.1 ± 1.1	9.5 ± 5.5

Abbreviation: aTSC, apparent tissue sodium content.

## 4 Discussion

aTSC values and ^1^H T_2_* relaxation times were successfully determined in patients with patellar tendinopathy and in healthy controls. Significant differences in both aTSC values and ^1^H T_2_* relaxation times were observed between patients and controls. Both the intra‐ and inter‐reader ICC values demonstrated excellent reliability, and measurements after repositioning were reproducible, with deviations below 10% across the whole tendon.

Sodium relaxation times were successfully determined in three healthy volunteers, enabling a relaxation correction of the aTSC values. Thus far, tendon sodium relaxation times have only been published for the Achilles tendon [16]. Compared with the patellar tendon, the Achilles tendon has a lower T_1_ relaxation time of 20.4 ± 2.4 ms. The T_2l_* and *p*_*s*_ values are similar at 13.9 ± 0.8 ms and 31.6 ± 2.6%, respectively. However, the T_2s_* value in the patellar tendon is twice as long compared with the Achilles tendon at 1.4 ± 0.4 ms [16]. Differences in relaxation times between the tendons could stem from varying physical demands and resulting structural differences. The Achilles tendon has to provide shock absorption, whereas the patellar tendon functions more like a ligament [43]. The relaxation times and *p*_*s*_ values of the reference phantoms are in line with previous studies [16].

The chosen PVC was the recently introduced eSTC, because it has been shown to be robust in combination with the strongly inhomogeneous fields of surface coils, and it was originally developed for the use with tendons [24]. Additionally, the sodium distribution of tendinopathic tendons can be highly inhomogeneous (e.g., Figure 4b), and this distribution is better preserved with the eSTC than with methods that assume a constant signal in each tissue, such as the more widely used geometric transfer matrix method [24, 28]. The eSTC has so far not been widely applied to sodium data, but our results indicate that it is robust among different readers and reproducible after repositioning.

The aTSCs of the healthy volunteers were relatively evenly distributed throughout the tendon, with slightly elevated values at the tendon endpoints. This finding is consistent with the elevated GAG concentration in healthy tendons at the endpoints [44]. Similarly, the aTSCs in the proximal and distal sections were higher than in the middle section in patients; however, this difference was not significant. Although tendinopathy is more prevalent at the endpoints of the patellar tendon, especially at the proximal part [45], three out of seven patients had structural alterations throughout the whole tendon, which was discernible on the clinical MRI images. The severity of the tendon's structural changes varied from minimal to severe among patients, explaining the higher standard deviations in both aTSC and ^1^H T_2_* values. Due to the small sample size, we did not correlate these categories of severity with aTSC or ^1^H T_2_* values.

To date, sodium MRI has rarely been applied to investigate tendons in general or patellar tendons in particular. To the best of our knowledge, Marik et al. [17] were the only previous researchers to apply sodium MRI to the patellar tendon. However, they did not calculate aTSC values but provided normalized signal intensities, which are difficult to compare with our results. They found a significant increase in signal intensity in patients with type 1 diabetes compared with volunteers, with patients exhibiting a signal intensity that was about 25% higher than in healthy controls. This increase is lower than in our case, yet their patients did not present morphological changes [17], in contrast to our tendinopathy patients, implicating the severity of disease in our patients might be higher.

We previously calculated aTSC values in the Achilles tendon, resulting in a mean value of 82.2 mM [16], which is considerably higher than the values observed in the present study. However, these values were obtained using a PVC algorithm that assumes zero sodium signal in the immediate surroundings of the structure, which is not the case for Achilles tendons [24]. The results from our previous study introducing the eSTC [24] are more comparable. The aTSCs of the Achilles tendon with application of the eSTC were 17.0 ± 11.6 and 22.1 ± 13.3 mM for the two healthy volunteers at 2 mm [3] isotropic resolution. This is consistent with our results in the patella tendon. Furthermore, Juras et al. determined the sodium SNR in the Achilles tendon, finding an SNR of 4.9 ± 2.1 in healthy controls and 9.3 ± 2.3 in patients with tendinopathy. While SNR is suboptimal for comparisons of different studies, because it depends strongly on imaging parameters and hardware [16], the scale of the signal increase between controls and patients was similar to our findings.

Previous studies have measured ^1^H T_2_* relaxation times in the patellar tendon and using both monoexponential and biexponential fits to separate individual water components [29, 30, 31, 46, 47, 48]. To keep the measurement times reasonable, we measured only four echo times, thus limiting our analysis to monoexponential fits. In literature, monoexponential ^1^H T_2_* values are generally longer in patients with tendinopathy than in healthy volunteers, with reported values ranging from about 1.03 ms^48^ to 2.0 ms^30^ in healthy tendons and about 1.4 ms^48^ to 6.43 ms^47^ in patients. Our results are within this range.

Both aTSC and ^1^H T_2_* values were altered in patients with tendinopathy. However, sodium MRI may be more sensitive to early changes, before the structural changes affect the water content [49]. This is supported by findings of previous studies, such as a stronger correlation with GAG content [12] or sensitivity to effects of medication [15].

## 5 Limitations

This study has several limitations. First, no individual B_1_ correction was applied to the sodium data. This was omitted to maintain clinically feasible scan times for human volunteers. Additional B_1_ maps for each volunteer would greatly increase measurement time if the double‐angle method [37, 50] was used or would reduce SNR in case of the Bloch–Siegert shift method [51]. Because the sodium SNR of tendons is inherently low [14], a further decrease would negatively impact the quality of the measurement. Instead, a coil sensitivity correction using a phantom measurement was applied. However, correcting the coil sensitivity with a homogeneous NaCl solution phantom can also only approximate the true sensitivity distribution in the knee, as it has different coil loading and dielectric properties [24, 52]. The inherently low signal was also the reason for the relatively short TR of the sodium protocol. To achieve an acceptable SNR, a high number of projections was required, while TR had to be reduced to keep the scan times reasonable [16]. Therefore, our measurements were affected by T_1_‐relaxation weighting, which was corrected using the relaxation correction described above [38]. Ideally, the relaxation times would be measured individually for each volunteer and patient. However, the long scan times of the relaxation protocols made this impractical. Thus, relaxation measurements were limited to three healthy volunteers and the average relaxation times were used for correction.

Furthermore, the relaxation time measurements themselves have limitations. The T_1_ measurement has a low number of TRs, with the longest TR being only 30 ms. Reducing the number of projections to facilitate additional data points within an acceptable total scan time would have resulted in insufficient SNR at short TRs, thereby compromising curve fitting in this range. Similarly, the ^1^H T_2_* relaxation time measurements had a low number of data points in order to keep the measurement time short, with only four TEs. While additional data points would be preferable for stable fitting, previous studies have found differences in ^1^H T_2_* between tendinopathy patients and healthy controls with four TEs [31, 48]. Finally, the number of patients with patellar tendinopathy was relatively low. Future studies with larger patient cohorts are needed to enable more robust statistical evaluations to further validate the present findings.

## 6 Conclusion

We successfully adapted sodium MRI for application in the patellar tendon by measuring the patellar tendons sodium relaxation times for relaxation correction and applying PVC. Consequently, aTSC values and ^1^H T_2_* relaxation times could be determined in healthy controls and patients with patellar tendinopathy. Both parameters were significantly increased in patients with tendinopathy, validating aTSC as an additional quantitative marker of patellar tendinopathy. Therefore, once sodium MRI has been further developed to meet clinical requirements, it could be a promising method of detecting biochemical tendon alterations.

## Author Contributions

**Rika Möller:** conceptualization, data curation, formal analysis, investigation, methodology, project administration, software, validation, visualization, writing – original draft preparation. **Julia Jurisic:** data curation, formal analysis, investigation, validation, writing – review and editing. **Lukas R. Drewes:** formal analysis, investigation, validation, writing – review and editing. **Alexandra Ljimani:** formal analysis, validation, supervision, writing – review and editing. **Hans‐Jörg Wittsack:** conceptualization, project administration, methodology, supervision, writing – review and editing. **Armin M. Nagel:** methodology, resources, software, writing – review and editing. **Gerald Antoch:** resources, supervision, writing – review and editing. **Lena M. Wilms:** conceptualization, formal analysis, methodology, validation, writing – review and editing. **Anja Müller‐Lutz:** funding acquisition, project administration, supervision, writing – review and editing. **Benedikt Kamp:** conceptualization, methodology, project administration, software, validation, supervision, writing – review and editing.

## Funding

Rika Möller was supported by the Jürgen Manchot Foundation, Düsseldorf, Germany.

## Conflicts of Interest

The authors declare no conflicts of interest.

## Data Availability

The data that support the findings of this study are available from the corresponding author upon reasonable request.
